# Commentary: Prediction of problem gambling by demographics, gaming behavior and psychological correlates among gacha gamers: A cross-sectional online survey in Chinese young adults

**DOI:** 10.3389/fpsyt.2025.1576323

**Published:** 2025-08-25

**Authors:** Leon Y. Xiao, Nick Ballou, Charlotte Eben

**Affiliations:** ^1^ School of Creative Media, City University of Hong Kong, Hong Kong, China; ^2^ beClaws.org, London, United Kingdom; ^3^ Oxford Internet Institute, University of Oxford, Oxford, United Kingdom; ^4^ Centre for Gambling Research, University of British Columbia, Vancouver, Canada; ^5^ Biopsychology Lab, University of Cologne, Cologne, Germany

**Keywords:** loot boxes, gacha, problem gambling, measurement schmeasurement, survey question design, scale construction and development

## 1 Introduction

Modifying core items created a novel construct wrongly identified as measuring problem gambling. Tang et al. (1) purported to report that ‘problem gambling’, as they defined it, is associated with monetary spending on video game mechanics that involve randomisation (e.g., gacha mechanics (2, 3) and loot boxes (4)) amongst young Hong Kong players of games containing such mechanics. At face value, that assertion is not surprising, as a consistent line of research has previously established that relationship in Western countries (5–7), which has been relied upon in policymaking (8, 9). Research conducted subsequent to Tang et al. in Mainland China has also replicated the relationship (10).

However, unfortunately, the study of Tang et al. suffered from a fundamental flaw. That study did not, in fact, measure ‘problem gambling’ as traditionally defined because Tang et al. significantly modified the measurement scale. Instead, Tang et al. measured only ‘problematic participation in gacha mechanics’ and proved that various relationships existed between that construct and other variables, which arguably is less meaningful (11, 12). The development of that new scale lacked transparency and evidence of reliability and validity. It is unclear whether that new measure is related to ‘problem gambling’ and, if so, to what extent. In any case, it is not, and cannot be used as, a direct replacement without further validation.

Tang et al. could not, in fact, have been able to report on any issues concerning ‘problem gambling’ because by definition, they did not measure ‘problem gambling’. They should not have claimed to have been able to test or comment on any psychological relationships concerning ‘problem gambling’. The current framing of that paper and its conclusions is misleading to readers whose attention is not specifically drawn to this highly significant measurement modification. This commentary intends to correct that misimpression. Simply put, Tang et al. did not prove what their title suggested. That paper must be treated with due caution and considered for exclusion from meta-analyses.

## 2 Discussion

Specifically, when purporting to measure ‘problem gambling’, Tang et al. used a significantly modified version of the Chinese version of the Problem Gambling Severity Index (PGSI). The PGSI was originally created in English (13) but has since been translated and validated in Chinese (14). Therefore, the use of the Chinese language version of the PGSI instead of the English version is not at issue. In fact, the Chinese PGSI has been used with success in other loot box studies in China (10, 15).

The problem was that Tang et al. modified the wording of the PGSI beyond a mere translation: references to ‘gambling [賭博]’ were all converted to ‘spending money on gacha mechanics [課金抽蛋]’, as the authors revealed in a personal response to LX’s query and as shown in **Table 1**. This modification was disclosed at page 4 of the original paper only very vaguely and not sufficiently prominently (‘As PGSI-C was originally developed for screening general gambling activities, the authors had modified a few words on some items to fit the context of gacha games’) (1). All modifications made should have been fully disclosed in detail.

**Table 1:** The Problem Gambling Severity Index compared to the Tang et al.’s problematic participation in gacha mechanics scale (with changes marked with red italics).

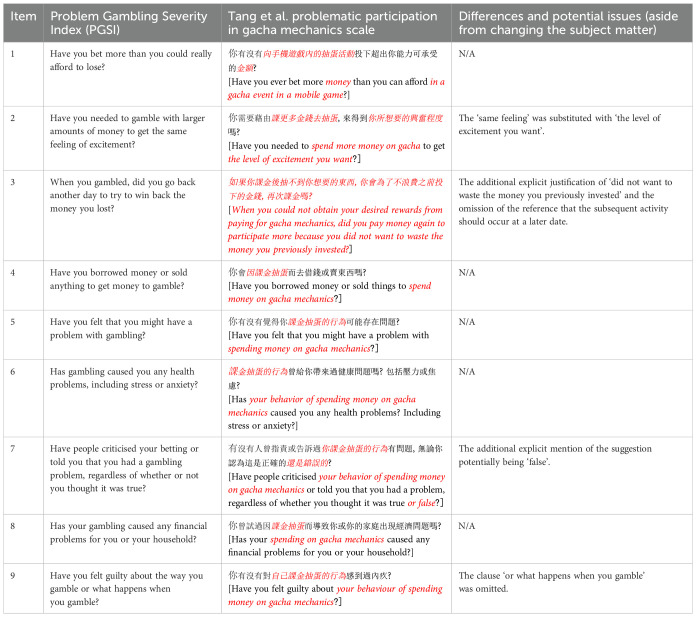

In theory, making such modifications to create a new scale is not problematic in and of itself, although when not done properly and transparently, it might constitute so-called ‘measurement schmeasurement’ or ‘questionable measurement practices’ that ‘raise doubts about the validity of the measures, and ultimately the validity of study conclusions’ (16, p.456). Other constructs, whose developments were much better detailed, have sought to measure potentially problematic engagement with loot boxes, e.g., the ‘Risky Loot Box Index’ (RLI) (10, 17–19) and the ‘Problematic Use of Loot Boxes Questionnaire’ (PU-LB) (20, 21). However, Tang et al.’s continued representation of the modified scale as if it measured ‘problem gambling’ as traditionally understood was misleading because following the modification, the scale instead measured ‘problematic participation in gacha mechanics’—a completely different construct. The modification effectively created a new and, importantly, *unvalidated* problematic gacha engagement scale more comparable to the RLI (10, 17, 18). This means that Tang et al. misrepresented their construct of ‘problematic participation in gacha mechanics’ as a measure of ‘problem gambling’ throughout the paper, from title to abstract, through the results, and into the conclusion.

Other substantive changes were also made to the PGSI. For example, the third item of the PGSI was not just slightly amended by having a few words modified but completely replaced, causing its meaning to change. The original question was: ‘When you gambled, did you go back another day to try to win back the money you lost?’ (13). The third question in the modified scale used by Tang et al. was: ‘When you could not obtain your desired rewards from paying for gacha mechanics, did you pay money again to participate more because you did not want to waste the money you previously invested?’ It is obvious that Tang et al. tried to replicate a similar sentiment of ‘loss chasing’ in both contexts (22), but there is a conceptual difference between a) more actively wanting to win back past losses and b) more passively not wanting to waste previous investments referenced by Tang et al.’s new item, which is more akin to entrapment and the sunk cost fallacy (23). The explicit reference to wasting previous investments could have been omitted in the modified scale, which could have just asked whether the participant tried to win the desired reward again by spending more money at a later date. Again, the question that was asked may have been perfectly acceptable for measuring the underlying behaviour. However, there was a lack of transparent disclosure of the modification and the justifications thereof, which casts doubt on the reliability of the measurement construction, its validity, and resultant findings (16).

## 3 Conclusion

Research conducted in Mainland China after Tang et al. has confirmed that spending on loot boxes is associated with problem gambling as traditionally understood (10), thus alleviating concerns that the relationship may not be replicable beyond Western samples (15, 24). Nonetheless, it must be clarified that Tang et al. did not measure ‘problem gambling’ but ‘problematic participation in gacha mechanics’ instead, which is a possibly related but substantially different construct. Readers must be informed that they ought to approach Tang et al. by mentally amending all references to ‘problem gambling’ in the paper (including title and abstract) to the new, unvalidated construct of ‘problematic participation in gacha mechanics’ instead, which is incredibly burdensome. Tang et al. must be corrected to ensure the accuracy and integrity of the scientific record. Attempts should also be made to validate the modified scale used by Tang et al. to better understand how that new construct is related to the traditional ‘problem gambling’ construct and whether the modified scale may have future utility as an alternative to the RLI (10, 17–19) and the PU-LB (20, 21).

## Author’s note

LX plays and enjoys video games and broadly views the activity very positively, except for certain aspects (e.g., monetisation) that he believes should be subject to more scrutiny. In terms of LX’s personal engagement with loot boxes, he has played and continues to play video games containing loot boxes, such as *Hearthstone* (Blizzard Entertainment, 2014) until 2018 and *Genshin Impact* (miHoYo, 2020) and *Zenless Zone Zero* (miHoYo, 2024) since their initial release. He, therefore, engaged and continues to engage with non-paid loot boxes on a regular basis. However, he has never purchased any loot boxes with real-world money aside from negligible spending for research purposes, e.g., to confirm the presence of paid loot boxes.
